# Compliance With Protective Behavioral Recommendations in the Outbreak of COVID-19 Among People Working in the Urban-Based Informal Economy in Southern Ethiopia

**DOI:** 10.3389/fpubh.2021.716814

**Published:** 2021-08-06

**Authors:** Bewunetu Zewude, Belayneh Melese, Tewodros Habtegiorgis, Mihret Tadele, Weynishet Solomon

**Affiliations:** ^1^Department of Sociology, Wolaita Sodo University, Sodo, Ethiopia; ^2^Department of Civics and Ethical Studies, Wolaita Sodo University, Sodo, Ethiopia

**Keywords:** compliance, COVID-19, informal economy, washing hands, wearing mask

## Abstract

Regardless of the advocacies made by the media and numerous organizations about the need for preventing the spread of COVID-19, there still exists a gap as far as compliance to regular implementation of the preventive mechanisms within communities is concerned. The purpose of the present study was, therefore, to examine compliance to personal protective behavioral recommendations to contain the spread of COVID-19 among urban residents engaged in the informal economic activities in Wolaita Sodo town, Southern Ethiopia. A cross-sectional study design was used where quantitative data were collected through the survey research method. Three hundred and eighty-four participants of the urban-based informal economy were randomly selected and contacted in their own natural settings with an interviewer-administered questionnaire. Data were inserted into SPSS software for analysis that involved both descriptive and inferential statistics, including frequency and percentage distributions, binomial and multinomial logistic regressions. The results of the research indicated that only 35.4% of the respondents regularly wore a mask. In addition, 54.9% of the survey participants disclosed that they do not clean their hands with disinfectants after touching objects under circumstances where they cannot get access to water and soap. Moreover, the most commonly reported reason of respondents for non-compliance to regular wearing of a mask has been its inconvenience or discomfort (62.8%), followed by the need to appear indifferent because most people around them do not wear a mask (25.2%). Furthermore, experiences of the respondents of regularly wearing a mask are significantly associated with regular attendance of the media regarding the preventive mechanisms of COVID-19 (OR = 0.224; *P* < 0.001; 95%C.I: 0.109–0.460), knowledge of someone ever infected by COVID-19 (OR = 0.402; *P* < 0.05; 95%C.I: 0.190–0.851), the belief that COVID-19 causes a severe illness (OR = 0.444; *P* < 0.05; 95%C.I: 0.201–0.980), and perception of the likelihood of dying as a result of infection by COVID-19 (OR = 0.374; *P* < 0.01; 95% C.I: 0.197–0.711). The authors have found a low level of compliance to the recommended safety measures, especially wearing of masks. It is, therefore, important that continued efforts of raising awareness should be done by all the concerned bodies. Above all, urban safety net programs that aim at keeping such social groups at home, at least during the critical wave of the pandemic, should also be strengthened.

## Introduction

It is widely reported that the COVID-19 pandemic has entered a new stage with rapid spread in almost all the countries of the world claiming the lives of more than 3 million people (1). It is expected, and of course, practically observed in some parts of the world that the healthcare systems face shortages of personnel and medical equipment supplies to treat the critically ill during the pandemic (2). Therefore, countries worldwide are encouraged to assess the existing community engagement structures and to use community engagement approaches to support contextually specific, acceptable, and appropriate COVID-19 prevention and control measures (3, 4). In addition, all members of the society must understand and practice measures for self-protection and for the prevention of transmission of infection to others (5, 6).

In developing countries such as Ethiopia where access to COVID-19 vaccine to every citizen is difficult to achieve, the most important way to control the disease among the populations is regular hand washing, regular wearing of face masks, the use of disinfectants, and the prevention of contact with the face and mouth after interacting with the infected environment (7). In response to the outbreak and spread of COVID-19, many countries have been using a combination of containment and mitigation activities with the intention of delaying major flow of patients and decreasing the demand for hospital beds, including different levels of contact tracing and isolation; avoiding touching of eyes, nose, and mouth; routine cleaning and disinfection of the environment; wearing face mask; social distancing; preparation of health systems for an outpour of severely ill patients who require isolation, oxygen, and mechanical ventilation; strengthening health facility infection prevention and control, with special attention to nursing home facilities; and postponement or cancelation of large-scale public gatherings (5, 6).

Nevertheless, regardless of the advocacies made by the media and numerous organizations about the need for preventing the spread of the pandemic, including the various ways of transmission, there still exists a gap as far as compliance to regular implementation of the preventive mechanisms within communities is concerned (8). Although many countries have been taking different preventive measures, some of these interventions became ineffective in stopping or at least reducing the fast spread of the pandemic which continued claiming millions of lives (9). According to Reference (6), the indications for personal protective equipment should be based on many circumstances, including the setting, target audience, risk of exposure, and the transmission dynamics of the pathogen. In addition, the overuse or misuse of personal protective equipment will have a further impact not only on its effectiveness of preventing the pandemic, but also in inducing shortages on the supply, and hence on its accessibility to the rest of the population.

Findings of previous researches (10–13) undertaken in Ethiopia reveal a mix of both high and low knowledge of people toward COVID-19. A study by (8) found that more than half (54.11%) of the research participants have inadequate knowledge about the prevention of COVID-19 in which sex, age, residence, educational status, experiences of seeking information from healthcare workers, social media, and mass media are the factors affecting such knowledge among the study population. On the other hand, another study undertaken in Addis Ababa, Ethiopia, by Desalegn et al. (14) found a positive attitude held among most (60.7%) of the respondents.

The presence of a strong relationship between the perceived credibility of information sources about COVID-19 and the engagement of people in self-protective behaviors has been found by Lep et al. (15). According to the study, research participants tend to exhibit relatively lower trust on the information from mass media, social media, politicians, and political institutions whereas they tend to trust information from medical professionals and scientists. It was suggested that information about COVID-19 from credible sources would lead to better protective behaviors among the public, which will help to contain the spread of the pandemic. Tomczyk et al. (16) found high compliance (25%) with all recommendations; public compliance (51%), with high compliance regarding public but not personal behaviors; and low compliance (24%) with most behavioral recommendations that help to contain the infection of COVID-19 where low compliance is associated with male sex, younger age, and lower public stigma.

In addition to its impact on the health of workers and their families, COVID-19 adversely affects specific societal groups who are more vulnerable to labor market conditions, especially unprotected workers, including the self-employed, casual, and gig workers, who are likely to be disproportionately hit by the virus as they do not have access to paid or sick leave mechanisms, and are less protected by conventional social protection mechanisms and other forms of income smoothing (17). According to Reference (18), the populations most at risk are those that depend heavily on the informal economy, occupy areas prone to shocks, have inadequate access to social services or political influence, have limited capacities and opportunities to cope and adapt, and people who have no or limited access to technologies. In this regard, self-employed people working in areas having no regular income in Ethiopia, including youth and women involved in the informal economic activities, motorbike or taxi service providers, shoe-cleaners, lottery ticket sellers, and people engaged in all forms of daily labor can be considered as social groups most affected by the pandemic. Members of such social groups are double burdened not only because of their susceptibility to the disease but also that their livelihood is more likely to be severely affected by the pandemic during periods of economic and social lockdowns. The objective of the present research was, therefore, to explore the patterns of compliance to the use of protective devices (non-pharmaceutical interventions) to contain the spread of COVID-19 among the urbanites working in the informal economies in Southern Ethiopia.

## Methods and Materials

### Research Design

A cross-sectional study was carried using quantitative research approach. Through the survey method, quantitative data were collected from a sample of people working in the urban-based informal economy.

### Selection of Research Participants

The target group of the present research is the “urban poor” who are not able to earn a regular income as a result of working in the informal urban economy. Members of such social groups must always go to work to secure their daily bread. In doing so, these individuals should either stand amid or frequently move around relatively large gatherings of people, which puts them at a higher risk of infection by COVID-19. Moreover, living in the highly unpredictable tomorrow due to the irregular income does not allow members of this group to stay at home to protect themselves even during the critical periods of the pandemic. Therefore, it is important that they adhere to at least some of the personal protective behavioral recommendations such as wearing a mask and cleaning hands. Consequently, we have considered the inclusion of people commonly known to work in the urban informal economies including motorbike or taxi service providers, shoe-shine boys and girls, daily laborers, street vendors, lottery ticket sellers, and women working in the daily small markets (locally known as *gullit*). In order to determine the sample size, the researchers applied Cochran's (1977) formula for calculating the sample size of an unknown population as:

$$\begin{array}{l} n=\frac{z^{2}\;\times \;p\left(1-p\right)}{e^{2}}=1.96^{2}\times 0.5(1-0.5)/0.05^{2}=384 \end{array}$$

where *n* is the sample size, *z* is the selected critical value of desired confidence level, *p* is the estimated proportion of an attribute that is present in the population. Then, the final sample units were chosen on the basis of probability proportionate to the size sampling technique. The major inclusion criteria for sample selection were as follows: being the permanent resident of Wolaita Sodo town (having an urban residential background), employment or self-employment in the informal urban economy as a source of livelihood, and willingness to participate in the survey. From the 384 questionnaires used to collect data, 379 items were found to be correctly completed, making the response rate 98.7%.

### Research Method and Source of Data

Primary data were gathered mainly through the use of the survey research method. A self-administered questionnaire was prepared in English and then translated in to the local (*Amharic*) language. Then, data collectors, all of whom were instructors of sociology and civics and ethical education departments, were trained about the items in the questionnaire and all the things to be done in the process of data collection. Following this, a permission letter was secured from the concerned body of Wolaita Sodo University. Next, a pilot test was undertaken to prove the compatibility of the questionnaire on 5% of respondents having similar characteristics with the actual survey population. On the basis of feedback and comments obtained from the pilot survey, slight improvements were made to the instrument of data collection. The same questionnaire, having similar items, was duplicated according to the already determined sample size. Finally, the actual data collection activity was held in April and May 2021 under the supervision of the principal investigator. Respondents were told to freely raise any question that is unclear to them. The reliability of the instrument was checked through internal consistency of the response items using Cronbach's α, whereas both content and face validity measures were used to maintain their validity.

### Instrument Design

The questionnaire that served as an instrument of data collection in our research was partly taken from the studies of Lep et al. (15), Tomczyk et al. (16), and Zewude and Habtegiorgis (19) and adapted to the purpose at hand, whereas most other questions were developed by the researchers. The questionnaire mainly consists of two sections, namely, questions seeking the socio-demographic background of respondents and questions aimed at assessing the attitude of the research participants and experiences of implementing the personal protective behavioral recommendations. The first (background) section of the tool comprises variables such as sex, age, education, marital status, and the presence of children. In addition, this part also includes questions that aim at measuring perceptions of the respondent of COVID-19 and their previous contact or experience with the disease. For instance, respondents were asked if they believe that COVID-19 really exists in Ethiopia in general and the study area in particular, if they knew someone ever infected by or died of COVID-19, whether they believe that they can be infected by COVID-19, and their belief about whether they are likely to die from COVID-19, all with response categories of (1) Yes and (2) No.

The second section of the questionnaire aimed at measuring the experiences of the respondents in practicing the common personal protective behavioral recommendations. It involves questions such as “Do you regularly wear a mask?,” “Do you frequently wash your hands after touching objects?,” and “Where you cannot get access to water and soap, do you clean your hands with disinfectants after touching objects?” all with response categories of “yes” and “no”. Moreover, the section also includes questions that intend to assess the extent to which respondents believe in the effectiveness of the protective behavioral recommendations. For instance, we have asked respondents “Do you believe that a mask can prevent the transmission of COVID-19?” and “Do you believe that cleaning hands immediately after touching any object can prevent COVID-19?” with response categories of “yes” and “no”. Furthermore, with the expectation of non-compliance to the behavioral recommendations, potential reasons for such non-compliance were also included in the section.

### Method of Data Analysis

The questionnaires returned from the field were first checked against completeness. The correctly completed ones were then inserted into a statistical package for social sciences software. Data generated from the software were presented using both descriptive and inferential statistical tools. Data analysis was conducted using statistical techniques, including percentages, frequency distributions, and logistic regression analysis. The descriptive statistical techniques were mainly used to present data regarding the frequency and percentage distribution of responses pertaining to the socio-demographic characteristics of the respondents, level of compliance to the protective behavioral recommendations, reasons for non-compliance, and beliefs about the protective behavioral recommendations. The logistic regression test was used to examine the association between the socio-demographic characteristics of respondents and their compliance to the protective behavioral recommendations. In this case, independent variables having a significance level ≤0.05 were considered to be significantly associated with the dependent variable, and those having a significance level >0.05 were considered as not significantly associated.

## Results

The mean age of respondents has been found to be 24.6 (*SD* = 6.87). According to the data presented in Table 1, 57.5% of research participants are men and 42.5% are women. Furthermore, the educational background of the respondents reveals that most (29.6%) have attended secondary level education, followed by primary level education (25.3%), BA/BSc degree (16.9%), and college diploma (15.3%). It is also found that 59.1% of respondents have never been married and 35.9% are married. In addition, 66.5% of the survey participants did not have children during the time of data collection. Moreover, the majority (68.1%) of respondents disclosed that they regularly attend any media regarding the preventive mechanisms of COVID-19. It is also found that 72.3% of research participants believe that COVID-19 really exists in Ethiopia, whereas 56.5% of them believe that COVID-19 exists in the study area. Above all, most (79.9%) of the respondents disclosed that there is no one they know who was ever infected by COVID-19 and 84.2% replied that they do not know anyone who died of COVID-19. Data have also shown that 55.4% of respondents do not think they will be infected by COVID-19, whereas 56.5% of them perceive that they are not likely to die if infected by COVID-19. Importantly, 69.1% of respondents think that COVID-19 causes a severe illness.

**Table 1:** Socio-demographic background of respondents.

**Variables**	**Categories**	**Frequency (%)**
Sex	Male	218 (57.5%)
	Female	161 (42.5%)
Educational status	Never attended school	49 (12.9%)
	Primary (1–8) level	96 (25.3%)
	Secondary (9-12) level	112 (29.6%)
	College diploma	58 (15.3%)
	BA/BSc degree	64 (16.9%)
Marital status	Never married	224 (59.1%)
	Married	136 (35.9%)
	Divorced	12 (3.2%)
	Widowed	7 (1.8%)
Do you have children?	Yes	127 (33.5%)
	No	252 (66.5%)
Do you regularly attend media regarding the preventive mechanisms of COVID-19?	Yes	258 (68.1%)
	No	121 (31.9%)
Do you believe that COVID-19 really exists in Ethiopia?	Yes	274 (72.3%)
	No	105 (27.7%)
Do you believe that COVID-19 really exists in Wolaita Sodo town (study area)?	Yes	214 (56.5%)
	No	165 (43.5%)
Do you know someone ever infected by COVID-19?	Yes	76 (20.1%)
	No	303 (79.9%)
Do you know someone died of COVID-19?	Yes	60 (15.8%)
	No	319 (84.2%)
Do you think that you can be infected by COVID-19?	Yes	169 (44.6%)
	No	210 (55.4%)
Do you think that COVID-19 causes severe illness?	Yes	262 (69.1%)
	No	117 (30.9%)
Do you think that you are likely to die if infected by COVID-19?	Yes	165 (43.5%)
	No	214 (56.5%)
	Total	379 (100%)

### Compliance With Preventive Behavioral Recommendations

Table 2 presents the frequency and percentage distributions of responses regarding the compliance of the respondents to the personal protective behavioral recommendations to contain the spread of COVID-19. Accordingly, though 70.4% of the research participants believe that a mask is effective in terms of preventing the transmission of COVID-19, only 35.4% of respondents reported to regularly wearing the mask. Furthermore, 73.3% of respondents believe that cleaning hands immediately after touching any object can prevent COVID-19 and 57% of them disclosed that they frequently wash their hands after touching objects. Moreover, 54.9% of respondents reported that they do not clean their hands with disinfectants after touching objects under circumstances where they cannot get access to water and soap.

**Table 2:** Distribution of compliance to personal protective behavioral recommendations.

**Variables**	**Categories**	**Frequency (%)**
Do you believe that a mask can prevent the transmission of COVID-19?	Yes	267 (70.4%)
	No	112 (29.6%)
Do you regularly wear a mask?	Yes	134 (35.4%)
	No	245 (64.6%)
Do you believe that cleaning hands immediately after touching any object can prevent COVID-19?	Yes	277 (73.3%)
	No	102 (26.9%)
Do you frequently wash your hand after touching objects?	Yes	216 (57%)
	No	163 (43%)
Where you can't get access to water and soap, do you clean your hand with disinfectants after touching objects?	Yes	171 (45.1%)
	No	208 (54.9%)
	Total	379 (100%)

According to the data presented in Table 3, the most commonly reported reason of respondents for non-compliance to regular mask wearing has been its inconvenience or discomfort (62.8%), followed by the need to appear indifferent because most people around them do not wear a mask (25.2%), the belief that COVID-19 does not really exist (22.5%), the belief that COVID-19 is not that serious concern in the work or residential area of the respondents (19.8%), and perceptions of lower risk of infection by COVID-19 (19.8%). In addition, lack of access to water and soap (58.3%), the belief that COVID-19 does not really exist (35.4%), the belief that COVID-19 is not that serious concern in the work or residential area of the respondents (25.1%), and reasons related to religion (14.3%) have constituted the main reasons that respondents do not frequently wash their hands after touching objects. Above all, lack of access to hand sanitizer/alcohol (58.5%), the belief that COVID-19 does not really exist (28.5%), the belief that COVID-19 is not that serious concern in the work or residential area of the respondents (25.1%), perceptions of lower risk of infection by COVID-19 (20.8%), the absence of belief that cleaning hands with disinfectants prevents infection (19.8%), and lack of adequate information (12.1%) have been found to be the main reasons for not cleaning hands with disinfectants after touching objects among the respondents.

**Table 3:** Reasons of the respondents for non-compliance to personal protective behavioral recommendations.

**Reasons**	**N (%)**	**% of Cases**
**Reasons for not regularly wearing a mask**		
Due to its inconvenience/discomfort	162 (30.9%)	62.8%
I just want to appear indifferent because most people around me do not wear mask	65 (12.4%)	25.2%
I can't afford to buy one because of its cost	19 (3.6%)	7.4%
I don't believe that a mask can prevent infection	39 (7.4%)	15.1%
I don't believe that COVID-19 really exists	58 (11.0%)	22.5%
I believe that I have adequate natural immunity	6 (1.1%)	2.3%
I believe that I am not at risk of being infected by COVID-19	51 (9.7%)	19.8%
I believe that I can easily withstand the illness if infected by the disease	6 (1.1%)	2.3%
Lack of adequate information about it	14 (2.7%)	5.4%
Reasons related to belief/religion	33 (6.3%)	12.8%
COVID-19 is not that serious concern in my work or residential area	51 (9.7%)	19.8%
Other reasons	17 (3.2%)	6.6%
No reason	4 (0.8%)	1.6%
Total	525 (100.0%)	203.5%
**Reasons for not frequently washing hands after touching objects**		
Lack of access to water/soap	102 (29.3%)	58.3%
I avoid touching of objects from the outset	7 (2.0%)	4.0%
I don't believe that COVID-19 really exists	62 (17.8%)	35.4%
I don't believe that washing hands can prevent infection	23 (6.6%)	13.1%
Because I use chemical disinfectants instead of washing my hands	20 (5.7%)	11.4%
I believe that I have adequate natural immunity	6 (1.7%)	3.4%
I believe that I am not at risk of being infected by COVID-19	34 (9.8%)	19.4%
I believe that I can easily withstand the illness if infected by the disease	1 (0.3%)	0.6%
Lack of adequate information	11 (3.2%)	6.3%
Reasons related to belief/religion	25 (7.2%)	14.3%
COVID-19 is not that serious concern in my work or residential area	44 (12.6%)	25.1%
Other reasons	7 (2.0%)	4.0%
No reason	6 (1.7%)	3.4%
Total	348 (100.0%)	198.9%
**Reasons for not cleaning hands with disinfectants after touching objects**		
Lack of access to hand sanitizer/alcohol	121 (30.3%)	58.5%
I avoid touching of objects from the outset	13 (3.3%)	6.3%
I don't believe that COVID-19 really exists	59 (14.8%)	28.5%
I don't believe that cleaning hands with disinfectants prevents infection	41 (10.3%)	19.8%
I believe that I have adequate natural immunity	5 (1.3%)	2.4%
I believe that I am not at risk of being infected by COVID-19	43 (10.8%)	20.8%
I believe that I can easily withstand the illness if infected by the disease	5 (1.3%)	2.4%
Lack of adequate information	25 (6.3%)	12.1%
Reasons related to belief/religion	21 (5.3%)	10.1%
COVID-19 is not that serious concern in my work or residential area	52 (13.0%)	25.1%
Other reasons	10 (2.5%)	4.8%
No reason	4 (1.0%)	1.9%
Total	399 (100.0%)	192.8%

Table 4 presents data pertaining to the extent (frequency) to which respondents comply with the major protective behavioral recommendations to contain the spread of COVID-19. It is found that 64.9% of respondents reported to occasionally wearing a mask and 9.2% said they never wore a mask. Moreover, 51.7% of respondents occasionally engage in frequent hand hygiene practices whereas 37.7% reported to regularly keep hand hygiene. Furthermore, 42.5% of the survey participants replied that they never keep adequate physical distance during public gatherings. Around 54.4% of the respondents disclosed habits of covering mouth and nose with flexed elbow or tissue when coughing or sneezing. In addition, 57.8% of the research participants never stay at home when they are feeling sick or symptomatic of COVID-19. It is also shown that 36.1% always participate in mass events (attending religious/cultural rituals, business/work meetings, social gatherings, etc.) while 58.6% of them do it occasionally. Above all, 34.3% reported to always shake hands, 32.2% of respondents disclosed that they would always touch their face, and 62.2% of them do the same occasionally. Occasional eating of raw/fresh foods (raw meat, vegetables, etc.) before cooking or washing has also been reported among 57% of the research participants.

**Table 4:** Extent of practices and compliances with the major protective behavioral recommendations.

**Variables/questions**	**Categories of responses**	**Frequency (%)**
Wearing a (medical) mask	Always	98 (25.9%)
	Occasionally	246 (64.9%)
	Never	35 (9.2%)
Frequent hand hygiene (use hand sanitizers, recurrent washing of hands with soaps, etc.)	Always	143 (37.7%)
	Occasionally	196 (51.7%)
	Never	40 (10.6%)
Keeping adequate (up to 2 meters) physical distance during gatherings	Always	34 (9%)
	Occasionally	184 (48.5%)
	Never	161 (42.5%)
Keeping your mask clean (by washing or replacing it with a new one)	Always	176 (46.4%)
	Occasionally	162 (42.7%)
	Never	41 (10.8%)
Covering mouth and nose with flexed elbow or tissue when coughing or sneezing	Always	136 (35.9%)
	Occasionally	206 (54.4%)
	Never	37 (9.8%)
Staying at home when feel sick/symptomatic	Always	42 (11.1%)
	Occasionally	118 (31.1%)
	Never	219 (57.8%)
Shaking hands with any person	Always	130 (34.3%)
	Occasionally	184 (48.5%)
	Never	65 (17.2%)
Touching one's face (i.e., eyes, nose, and mouth)	Always	122 (32.2%)
	Occasionally	232 (61.2%)
	Never	25 (6.6%)
Eating raw/fresh foods (raw meat, vegetables, etc.) before cooking or washing	Always	23 (6.1%)
	Occasionally	216 (57%)
	Never	140 (36.9%)
Participating in mass events (attending religious/cultural rituals, business/work meetings, social gatherings, etc.)	Always	137 (36.1%)
	Occasionally	222 (58.6%)
	Never	20 (5.3%)
Total		379 (100%)

### Factors Affecting Compliance to Preventive Behavioral Recommendations

The data presented in Table 5 show that the experiences of the respondents of regular mask wearing are significantly associated with regular attendance of the media regarding the preventive mechanisms of COVID-19 (OR = 0.224; *P* < 0.001; 95% C.I: 0.109–0.460), knowledge of someone ever infected by COVID-19 (OR = 0.402; *P* < 0.05; 95% C.I: 0.190–0.851), the belief that COVID-19 causes severe illness (OR = 0.444; *P* < 0.05; 95% C.I: 0.201–0.980), and perception of the probability of dying as a result of infection by COVID-19 (OR = 0.374; *P* < 0.01; 95% C.I: 0.197–0.711).

**Table 5:** Binary logistic regression showing association between socio-demographic characteristics and practices of regular mask wear.

**Variables**	**Categories**	**Do you regularly wear a mask?**			**Logistic Regression statistics**		
		**Yes**	**No**	**Total**	***P*-value**	**OR**	**95% C.I**.
Regular attendance of media	Yes	121	137	258	0.000^***^	0.224	0.109–0.460
	No	13	108	121			
Sex	Male	87	131	131	0.118	0.647	0.375–1.116
	Female	47	114	161			
Marital status	Never married	84	140	224	0.195	0.167	0.011–2.505
	Married	47	89	136			
	Divorced	2	10	12			
	Widowed	1	6	7			
Have children	Yes	46	81	127	0.257	0.571	0.217–1.504
	No	88	164	252			
Education	Never attended school	15	34	49	0.765	1.167	0.424–3.207
	Primary level	25	71	96			
	Secondary level	41	71	112			
	College diploma	24	34	58			
	BA/BSc degree	29	35	64			
Believe that COVID-19 exists in Ethiopia	Yes	118	156	274	0.890	0.939	0.385–2.288
	No	16	89	105			
Believe that COVID-19 exists in the study area	Yes	97	117	214	0.684	1.167	0.554–2.457
	No	37	128	165			
Knowledge of someone ever infected by COVID-19	Yes	44	32	76	0.017^*^	0.402	0.190–0.851
	No	90	213	303			
Know someone died of COVID-19	Yes	30	30	60	0.446	1.373	0.607–3.103
	No	104	215	319			
Think can be infected by COVID-19	Yes	95	74	169	0.082	0.565	0.297–1.074
	No	39	171	210			
Believe that COVID-19 causes severe illness	Yes	119	143	262	0.044^*^	0.444	0.201–0.980
	No	15	102	117			
Think die if infected by COVID-19	Yes	93	72	165	0.003^**^	0.374	0.197–0.711
	No	41	173	214			
Believe mask prevents COVID-19	Yes	120	147	267	0.276	0.641	0.288–1.427
	No	14	98	112			
Age					0.127	0.959	0.909–1.012

*** *P<0.001*,

** *P<0.01*,

* *P<0.05*.

*OR, odds ratio; C.I, confidence interval*.

Results of binary logistic regression coefficients presented in Table 6 reveal that the experiences of the research participants of regularly washing hands after touching objects are significantly associated with regular attendance of the media regarding the preventive mechanisms of COVID-19 (OR = 0.467; *P* < 0.05; 95% C.I: 0.256–0.852), having children (OR = 0.347; *P* < 0.05; 95% C.I: 0.137–0.878), the belief that frequently washing hands helps to prevent the transmission of COVID-19 (OR = 9.871; *P* < 0.001; 95% C.I: 4.245–22.954), and age (OR = 1.050; *P* < 0.05; 95% C.I: 1.003–1.100).

**Table 6:** Binary logistic regression showing association between the socio-demographic characteristics of the respondents and practices of frequent hand wash.

**Variables**	**Categories**	**Do you regularly wear a mask?**			**Logistic Regression statistics**		
		**Yes**	**No**	**Total**	***P*-value**	**OR**	**95% C.I**.
Regular attendance of media	Yes	168	90	258	0.013^*^	0.467	0.256–0.852
	No	48	73	121			
Sex	Male	119	99	218	0.097	1.575	0.921–2.694
	Female	97	64	161			
Have children	Yes	66	61	127	0.025^*^	0.347	0.137–0.878
	No	150	102	252			
Education	Never attended school	25	24	49	0.549	0.733	0.266–2.023
	Primary level	53	43	96			
	Secondary level	66	46	112			
	College diploma	29	29	58			
	BA/BSc degree	43	21	64			
Believe that COVID-19 exists in Ethiopia	Yes	174	100	274	0.289	1.604	0.669–3.844
	No	42	63	105			
Believe that COVID-19 exists in the study area	Yes	139	75	214	0.724	1.145	0.540–2.429
	No	77	88	165			
Know someone ever infected by COVID-19	Yes	52	24	76	0.361	0.693	0.316–1.522
	No	164	139	303			
Know someone died of COVID-19	Yes	40	20	60	0.993	1.004	0.435–2.314
	No	176	143	319			
Think can be infected by COVID-19	Yes	128	41	169	0.258	0.682	0.351–1.324
	No	88	122	210			
Believe that COVID-19 Causes serious illness	Yes	175	87	262	0.331	0.708	0.352–1.422
	No	41	76	117			
Think die if infected by COVID-19	Yes	122	43	165	0.360	0.738	0.386–1.413
	No	94	120	214			
Believe mask prevents COVID-19	Yes	184	83	267	0.464	0.739	0.329–1.661
	No	32	80	112			
Believe that frequently cleaning hands protects COVID-19	Yes	199	78	277	0.000^***^	9.871	4.245–22.954
	No	17	85	102			
Age					0.036^*^	1.050	1.003–1.100

*** *P<0.001*,

**P < 0.01,

* *P<0.05*.

*OR, Odds Ratio; C.I, Confidence Interval*.

The data presented in Table 7 show coefficients of multinomial logistic regression pertaining to the association between the socio-demographic characteristics of the respondents and the frequency by which they practice protective behavioral recommendations. Accordingly, it is found that respondents with a better educational status (OR = 0.035; *P* < 0.05; 95% C.I: 0.002–0.595), those who think that they can be infected by COVID-19 (OR = 58.942; *P* < 0.001; 95% C.I: 7.703–451.023), and those who believe that wearing mask prevents the transmission of COVID-19 (OR = 7.732; *P* < 0.005; 95% C.I: 1.332–44.893) tend to regularly wear a mask.

**Table 7:** Multinomial logistic regression coefficients.

**Wearing a (medical) mask**		***P*-value**	**OR**	**95% CI**
Regularly	Education	0.020^*^	0.035	0.002–0.595
	Think can be infected by COVID-19	0.000^***^	58.942	7.703–451.023
	Believe mask prevents COVID-19	0.023^*^	7.732	1.332–44.893
Occasionally	Education	0.047^*^	0.066	0.004–0.966
	Think can be infected by COVID-19	0.000^Y^	41.572	5.817–297.105
	Believe that COVID-19 Causes serious illness	0.019^*^	0.188	0.046–0.758
	Think die if infected by COVID-19	0.080^*^	0.279	0.067–1.167
	Believe mask prevents COVID-19	0.001^**^	15.989	3.318–77.061
Regularly	Frequent hand hygiene (use hand sanitizers, recurrent washing of hands with soaps, etc.)			
	Education	0.032^*^	0.133	0.021–0.839
	Think can be infected by COVID-19	0.020^*^	4.113	1.253–13.503
	Believe mask prevents COVID-19	0.008^**^	5.550	1.549–19.882
Occasionally	Marital status	0.000^***^	1.965	4.688–8.235
	Education	0.007^**^	0.080	0.013–0.498
	Believe mask prevents COVID-19	0.013^*^	4.216	1.359–13.080
Shaking hands with any person				
Regularly	Think can be infected by COVID-19	0.012^*^	0.278	0.102–0.759
	Believe mask prevents COVID-19	0.007^**^	0.146	0.036–0.590

*The reference category is: never*;

*** *P<0.001*,

** *P<0.01*,

* *P<0.05*.

*OR, Odds Ratio; C.I, Confidence Interval*.

Moreover, respondents with a better educational status (OR = 0.133; *P* < 0.05; 95% C.I: 0.021–0.839), who believe that wearing a mask prevents the transmission of COVID-19 (OR = 5.550; *P* < 0.001; 95% C.I: 1.549–19.882), and those who think that they are likely to be infected by COVID-19 (OR = 4.113; *P* < 0.05; 95% C.I: 1.253–13.503) are found to regularly engage in frequent hand hygiene (use hand sanitizers, recurrent washing of hands with soaps, etc.) practices. Above all, experiences of regularly shaking hands with someone have been found to be significantly associated with thinking that one is likely to be infected by COVID-19 (OR = 0.278; *P* < 0.05; 95% C.I: 0.102–0.759) and the belief that a mask can prevent the transmission of COVID-19 (OR = 0.146; *P* < 0.01; 95% C.I: 0.036–0.590).

## Discussion

During the period where COVID-19 pandemic has entered into a new stage of very fast spread across the world, the healthcare systems of most countries face shortages of personnel and medical equipment supplies to treat the critically ill (2). Therefore, all members of the society must understand and practice measures for self-protection and for prevention of transmission of infection to others (5, 6). Especially, in developing countries, such as Ethiopia, where access to COVID-19 vaccine to every citizen is difficult to achieve, the most important way to control the disease among the populations is the regular wearing of mask, regular hand washing, the use of disinfectants, and the prevention of contact with the face and mouth after interacting with the infected environment (7). Regardless of the advocacies made by the media and numerous organizations about the need for preventing the spread of the pandemic, including the various ways of transmission, there still exists a gap as far as compliance to regular implementation of the preventive mechanisms within communities is concerned (8). The purpose of the present study was to examine the experiences of compliance to personal protective behavioral recommendations (non-pharmaceutical interventions) to contain the spread of COVID-19 among urban residents working in the informal economic activities in Wolaita Sodo town, southern Ethiopia.

The results of the research indicated that only 35.4% of the respondents regularly wore a mask. In addition, 54.9% of survey participants disclosed that they do not clean their hands with disinfectants after touching objects under circumstances where they cannot get access to water and soap. Furthermore, 64.9% of respondents reported to occasionally wearing a mask and 9.2% said they never wore a mask. Moreover, 51.7% of respondents occasionally engage in frequent hand hygiene practices, whereas 37.7% reported to regularly keep hand hygiene. Furthermore, 42.5% of survey participants replied to never keep adequate physical distance during public gatherings. The finding of the present study that there is a low practice of wearing mask contradicts the findings of previous studies undertaken in other parts of the world. For instance, Zhong et al. (20) found that nearly all of the participants (98.0%) wore masks. In addition, Azlan et al. (21) revealed that most participants were taking precautions such as avoiding crowds (83.4%) and practicing proper hand hygiene (87.8%) in the week before the movement-control order started. He also found 51.2% practices of wearing face masks, which the authors regarded as a “less common” practice. Furthermore, Czeisler et al. (22) found widespread adherence to recommended COVID-19 mitigation strategies in the United States of America, in which 79.5% reported the behavior of always or often keeping ≥6 feet apart from others. Tomczyk et al. (16) found high compliance (25%) with all recommendations; public compliance (51%), with high compliance regarding public but not personal behaviors; and low compliance (24%) with most behavioral recommendations that help to contain infection of COVID-19, where low compliance is associated with male gender, younger age, and lower public stigma.

The findings of the study reveal a clear gap between the widely held belief about self-protection against COVID-19 and practices of implementing the recommended safety measures. For instance, 70.4% of respondents believe that a mask is effective in terms of preventing the transmission of COVID-19, and 73.3% of respondents believe that cleaning hands immediately after touching any object can prevent COVID-19. The findings of the present study that most research participants have a positive attitude toward the recommended protective measures are consistent with the findings of previous studies. A study undertaken in Addis Ababa, Ethiopia by Desalegn et al. (14) found a positive attitude held among most (60.7%) of the respondents. Another study Yonas Akalu et al. (13) stated that more than half (54.11%) of the research participants have inadequate knowledge about the prevention of COVID-19 in Ethiopia. Moreover, Alrubaiee et al. (23) concluded that the majority of health care providers in Yemen had adequate knowledge, optimistic attitude, moderate level of anxiety, and high-performance in preventive behaviors, 69.8, 85.10, 51.0, and 87.70%, respectively, toward COVID-19. Al-Hanawi et al. (24) also found that Saudi residents, especially women, have good knowledge, positive attitudes, and good practices toward COVID-19.

The results of our study have also revealed that the experiences of regular mask wearing of the respondents are significantly associated with regular attendance of the media regarding the preventive mechanisms of COVID-19, knowledge of someone ever infected by COVID-19, the belief that COVID-19 causes severe illness, and perception of the probability of dying as a result of infection by COVID-19. Moreover, it is also found that the experiences of research participants of regularly washing hands after touching objects is significantly associated with regular attendance of the media regarding the preventive mechanisms of COVID-19, having children, the belief that frequently washing hands helps to prevent the transmission of COVID-19, and age. Bashirian et al. (25) stated that threat and coping appraisal were predictors of protection motivation to conduct COVID-19 preventive behaviors. A study conducted by Shahnazi et al. (26) indicated that female sex, perceived barriers, perceived self-efficacy, fatalistic beliefs, perceived interests, and living in the city had the greatest preventive behaviors from COVID-19, respectively. Moreover, the presence of a strong relationship between perceived credibility of information sources about COVID-19 and engagement of peoples in self-protective behaviors has been found by Lep et al. (15). Tomczyk et al. (16) argued that low compliance to protective behavioral recommendations is associated with male gender, younger age, and lower public stigma. According to the health belief model, the key variables that determine the health behavior of an individual include perceived severity of an illness, whether the person thinks he is susceptible to certain ill-health conditions, perceived benefits that the person is likely to obtain as a result of engaging in a prohealth behavior, perceived barriers, the extent to which the individual is exposed to external events that prompt a desire to make a health change, and the self-efficacy of a person of bringing a health-related change (27–29).

## Conclusions

The main objective of the present research was to assess the level of compliance to the personal protective behavioral recommendations (non-pharmaceutical interventions) among the urban dwellers in Wolaita Sodo town working in the informal economic activities. The results of the study indicated that people engaged in the urban informal economy have a positive attitude toward the necessity of practicing protective behaviors that help to contain the spread of COVID-19. Nevertheless, we have found a low level of compliance to the recommended safety measures, especially wearing masks. The commonly identified reasons for the low compliance have been feelings of discomfort when wearing a mask, believing that COVID-19 does not exist at all or that it is not a serious concern in the residential or workplaces of the respondents, and perceptions of being at a lower risk of infection. Moreover, the study has also revealed that regular mask wearing among the research participants is significantly influenced by the experiences of regularly attending the media, knowledge of someone infected by COVID-19, the belief that COVID-19 causes a severe illness, and perception of the probability of dying as a result of infection by COVID-19. The findings of the study have great implications, especially when it comes to vulnerability and public health concerns. As in the case of the participants of the present study, urban dwellers whose livelihood predominantly relies on the informal economy characterized by irregular and unpredictable income should always remain outside of their homes in order to secure daily bread. These societal groups do not have the means to stay at home, even on witnessing the symptoms of COVID-19. This and many other characteristics of the group put them relatively at a higher risk of infection. It is therefore important that continued efforts of raising awareness should be done by all the concerned bodies. In addition, the government and other agencies in the area should devise mechanisms by which members of such group can get free access to the basic protective devices. Above all, urban safety net programs that aim at keeping such social groups at home at least during the critical wave of the pandemic should also be strengthened.

## Data Availability Statement

The raw data supporting the conclusions of this article will be made available by the authors, without undue reservation.

## Ethics Statement

The studies involving human participants were reviewed and approved by Wolaita Sodo University Ethics approval committee. Written informed consent for participation was not required for this study in accordance with the national legislation and the institutional requirements.

## Author Contributions

BZ reviewed literatures and wrote the research proposal. TH, MT, WS, and BM carried out fieldwork and drafted the manuscript. All contributed to the conception, analysis and writing of the manuscript. All authors read and approved the final manuscript.

## Conflict of Interest

The authors declare that the research was conducted in the absence of any commercial or financial relationships that could be construed as a potential conflict of interest.

## Publisher's Note

All claims expressed in this article are solely those of the authors and do not necessarily represent those of their affiliated organizations, or those of the publisher, the editors and the reviewers. Any product that may be evaluated in this article, or claim that may be made by its manufacturer, is not guaranteed or endorsed by the publisher.

## Abbreviations

SPSS: Statistical Package for Social Sciences
BA: Bachelor of Arts
BSc: Bachelor of Science
CSA: Central Statistical Agency
C.I: Confidence Interval
ERC: European Research Council
ILO: International Labor Organization
OR: Odds Ratio.
