# Supplementary material for: Compliance With Protective Behavioral Recommendations in the Outbreak of COVID-19 Among People Working in the Urban-Based Informal Economy in Southern Ethiopia
Source: Front Public Health. 2021 Aug 6;9:716814. doi: 10.3389/fpubh.2021.716814 (PMC8377721; doi:10.3389/fpubh.2021.716814)
Supplement: Supplementary file 1 [file Table_1.DOCX]

| No. | Variables/questions | Categories of responses | Answer  box |
| --- | --- | --- | --- |
|  | Sex | 1. Male 2. Female |  |
|  | Age | _______ | _______ |
|  | Marital status | 1. Never married 2. Married 3. Divorced 4. Widowed |  |
|  | Do you have children? | 1. Yes 2. No |  |
|  | Educational status | 1. Never attended school 2. Primary school (1-8) level 3. Secondary school (9-12) level 4. College diploma (10+3) 5. BA/BSc degree 6. MA/MSc degree & above |  |
|  | Do you regularly attend media regarding the preventive mechanisms of Covid-19? | 1. Yes 2. No |  |
|  | Do you think COVID-19 really exists in Ethiopia? | 1. Yes 2. No |  |
|  | Do you think that COVID-19 really exists in Wolaita Sodo town (study area)? | 1. Yes 2. No |  |
|  | Do you know someone ever infected by COVID-19? | 1. Yes 2. No |  |
|  | Do you know someone died of COVID-19? | 1. Yes 2. No |  |
|  | Do you think that you can be infected by Covid-19? | 1. Yes 2. No |  |
|  | Do you think that Covid-19 causes a serious illness? | 1. Yes 2. No |  |
|  | Do you think that you are likely to die if get infected by Covid-19? | 1. Yes 2. No |  |

| No. | Variables/questions | Categories of responses | Answer  box |
| --- | --- | --- | --- |
|  | Do you believe that a mask can prevent the transmission of Covid-19? | 1. Yes 2. No |  |
|  | Do you regularly wear a mask? | 1. Yes 2. No |  |
|  | If your answer for question number 1 is “no”, what is your reason?  (Multiple response is possible) | 1. Due to its inconvenience/discomfort 2. I just want to appear indifferent because most people around me do not wear mask 3. I can’t afford to buy one because of its cost 4. I don’t believe that a mask can prevent infection 5. I don’t believe that Covid-19 really exists 6. I believe that I have adequate natural immunity 7. I believe that I am not at risk of being infected by Covid-19 8. I believe that I can easily withstand the illness if infected by the disease 9. Lack of adequate information about it 10. Reasons related to belief/religion 11. Covid-19 is not that serious concern in my work or residential area 12. Other reasons 13. No reason |  |
|  | If your answer for question number 2 is yes, do you always maintain your mask clean? | 1. Yes 2. No |  |
|  | Do you believe that cleaning hands immediately after touching any object can prevent Covid-19? | 1. Yes 2. No |  |
|  | Do you frequently wash your hand after touching objects? | 1. Yes 2. No |  |
|  | If your answer is “no,” what is your reason/s?  (Multiple response is possible) | 1. Lack of access to water/soap 2. I avoid touching of objects from the outset 3. I don’t believe that Covid-19 really exists 4. I don’t believe that washing hands can prevent infection 5. Because I use chemical disinfectants instead of washing my hands 6. I believe that I have adequate natural immunity 7. I believe that I am not at risk of being infected by Covid-19 8. I believe that I can easily withstand the illness if infected by the disease 9. Lack of adequate information 10. Reasons related to belief/religion 11. Covid-19 is not that serious concern in my work or residential area 12. Other reasons 13. No reason |  |
|  | Where you can’t get access to water and soap, do you clean your hand with disinfectants after touching objects? | 1. Yes 2. No |  |
|  | If your answer above is no, what is your reason?  (Multiple response is possible) | 1. Lack of access to hand sanitizer/alcohol 2. I avoid touching of objects from the outset 3. I don’t believe that Covid-19 really exists 4. I don’t believe that cleaning hands with disinfectants prevents infection 5. I believe that I have adequate natural immunity 6. I believe that I am not at risk of being infected by Covid-19 7. I believe that I can easily withstand the illness if infected by the disease 8. Lack of adequate information 9. Reasons related to belief/religion 10. Covid-19 is not that serious concern in my work or residential area 11. Other reasons 12. No reason |  |

| No. | Variables/questions | Categories of responses | Answer  box |
| --- | --- | --- | --- |
|  | Wearing a (medical) mask | 1. Always 2. Occasionally 3. Never |  |
|  | Frequent hand hygiene (use hand sanitizers, recurrent washing of hands with soaps, etc.) | 1. Always 2. Occasionally 3. Never |  |
|  | Keeping adequate (up to 2 meters) physical distance | 1. Always 2. Occasionally 3. Never |  |
|  | Keeping your mask clean (by washing or replacing it with a new one) | 1. Always 2. Occasionally 3. Never |  |
|  | Covering mouth and nose with flexed elbow or tissue when coughing or sneezing | 1. Always 2. Occasionally 3. Never |  |
|  | Staying at home when feel sick/symptomatic | 1. Always 2. Occasionally 3. Never |  |
|  | Shaking hands with any person | 1. Always 2. Occasionally 3. Never |  |
|  | Touching one’s face (i.e., eyes, nose, and mouth) as much as possible | 1. Always 2. Occasionally 3. Never |  |
|  | Eating raw/fresh foods (raw meat, vegetables, etc.) before cooking or washing | 1. Always 2. Occasionally 3. Never |  |
|  | Participating in mass events (attending religious/cultural rituals, business/work meetings, social gatherings, etc.) | 1. Always 2. Occasionally 3. Never |  |

**
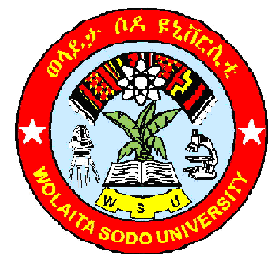
**

**የማሕበራዊ ሳይንስና ሥነ-ሰብ ኮሌጅ**

የሶሾሎጂ ት/ት ክፍል

**ውድ የዚህ መጠይቅ ተሳታፊዎች**: በቅድሚያ በዚህ ጥናት ላይ ለመሳተፍ ፍቃደኛ ስለሆናችሁ ከልብ እናመሰግናለን፡፡

ይህ መጠይቅ የተዘጋጀው፡ በወላይታ ሶዶ ዩኒቨርሲቲ፡ የማህበራዊ ሳይንስና ስነ-ሰብ ኮሌጅ፡ የሶሾሎጂ ት/ት ክፍል ባልደረባ በሆኑ ተመራማሪዎች ሲሆን፡ አገልግሎቱም፡ ከእናንተ ከጥናቱ ተሳታፊዎች ላይ የኮሮና በሽታን መከላከያ መንገዶችን አተገባበር በተመለከተ ያልዎትን ልምምድ ወይም ተሞክሮ በተመለከተ መረጃ ለመሰብሰብ ነው፡፡

ከእናንተ የሚሰበሰበው መረጃም፡ ***“****Patterns of Compliance with Self-Protective Behavioral Recommendations in the Outbreak of Covid-19 among People in the informal economy in Southern Ethiopia****”*** በሚል ርዕስ አሁን እየሰራን ላለነው የዳሰሳ ጥናት እንደ ግብዐት የሚያገለግል ይሆናል፡፡ የጥናቱ ዋነኛ ዓላማ፡ ቋሚ (ወርሃዊ) የመተዳደሪያ ገቢ የሌላቸው የህብረተሰብ ክፍሎች የኮሮና ቫይረስ በሽታን ለመከላከል የሚያስችሉ ዘዴዎችን በመተግበር ረገድ ያላቸውን ልምምድ እና ተሞክሮ ለመገንዘብ ነው፡፡

በመሆኑም፡ በዚህ መጠይቅ ውስጥ ለሚቀርብልዎ ጥያቄዎች ተአማኒነት ያላቸውን ምላሾች በሃቅ ላይ ተመስርተው እንዲሰጡን፡ በትህትና እንጠይቃለን፡፡

መጠይቁን ሲሞሉ: የሚከተሉትን ነጥቦች ልብ ይበሉ፡

- መጠይቁ በአጠቃላይ 4 ገፆች፣ 3 ክፍሎች እና 31 ጥያቄዎች እንዳለው ያረጋግጡ
- የመጠይቁ ወረቀት ላይ ስምዎን አይፃፉ፡ ይህም የመልሶችዎን ሚስጥራዊነት ለመጠበቅ ያስችላል
- መልስዎን 100% ሥርዓትና አግባብ ባለው መንገድ እንደምንይዘው እርግጠኛ ይሁኑ
- ***ሁሉንም ጥያቄዎች መመለስዎን ያረጋግጡ፣ ምንም ዓይነት ጥያቄ መዝለል በጥናቱ አጠቃላይ ሂደት እና ውጤት ላይ ተፅዕኖ ስለሚኖረው፡ እባክዎ***
- ምንም አይነት ጥያቄ ወይም አስተያየት ሲኖርዎ ወይም ተጨማሪ ማብራሪያ ካስፈለገ፡ ይጠይቁ ወይም በ0955325595 ወይም 0920643503 ላይ ይደውሉ
- ለሁሉም ጥያቄዎች መልስ ሰጥተው ከጨረሱ በኃላ፡ ወረቀቱን ለሰጥዎ ግለሰብ ይመልሱ፡፡

**ክፍል ፩፡ የተሳታፊዎችን መሠረታዊ መረጃዎች የሚዳስሱ ጥያቄዎች**

***መመሪያ፡ ከታች ለቀረቡት ጥያቄዎች፡ የእርስዎን ሃሳብ የሚገልፀው መልስ ላይ ካከበቡ በኃላ፡ መልስዎን የያዘውን ቁጥር/ሮች በመጨረሻው የመልስ መስጫ ሳጥን ውስጥ ያስቀምጡ***

| ቁ. | ጥያቄዎች/ተለዋዋጮች | የመልስ አማራጮች | የመልስ መስጫ ሳጥን |  |
| --- | --- | --- | --- | --- |
|  | ፆታ | 1. ወንድ 2. ሴት |  |  |
|  | ዕድሜ | _____ | ___ |  |
|  | የጋብቻ ሁኔታ | 1. ያላገባ/ች 2. ያገባ/ች 3. አግብቶ የፈታ/ች 4. ባል/ሚስት የሞተበ/ባት |  |  |
|  | ልጅ/ልጆች አለህ/ሽ? | 1. አዎ 2. የለኝም |  |  |
|  | የትምህርት ደረጃ | 1. ምንም ያልተማረ/ች 2. የመጀመሪያ ደረጃ (1-8) የተማረ/ች 3. ሁለተኛ ደረጃ (9-12) የተማረ/ች 4. ኮሌጅ ዲፕሎማ 5. የመጀመሪያ/ባችለር ዲግሪ 6. ሁለተኛ/ማስተርስ ዲግሪ እና ከዚያ በላይ |  |  |
|  | የኮሮና በሽታን በተመለከተ በተለያዩ ሚዲያዎች የሚተላለፉ የጥንቃቄ መልዕክቶችን ትከታተላለህ/ህ/ሽ? | 1. አዎ 2. አልከታተልም |  |  |
|  | የኮሮና በሽታ ኢትዮጵያ ውስጥ ያለ ይመስልሃል/ሻል? | 1. አዎ 2. አይመስለኝም |  |  |
|  | የኮሮና በሽታ ወላይታ ሶዶ ከተማ ውስጥ ያለ ይመስልሃል/ሻል? | 1. አዎ 2. አይመስለኝም |  |  |
|  | በኮሮና በሽታ የታመመ የቅርብ ዘመድ ወይም ጓደኛ ኖሮዎት ያውቃል? | 1. አዎ 2. የለም |  |  |
|  | በኮሮና በሽታ ታሞ የሞተ የቅርብ ዘመድ ወይም ጓደኛ ኖሮዎት ያውቃል? | 1. አዎ 2. የለም |  |  |
|  | የኮሮና በሽታ የሚይዝህ/ሽ ይመስልሃል/ሻል? | 1. አዎ 2. አይመስለኝም |  |  |
|  | የኮሮና በሽታ፡ ከባድ ህመም ያለው ይመስልሃል/ሻል? | 1. አዎ 2. አይመስለኝም |  |  |
|  | በኮሮና በሽታ ብትያዝ የምትሞት/የሚገድልህ/ሽ ይመስልሃል/ሻል? | 1. አዎ 2. አይመስለኝም |  |  |
| **ክፍል ፪፡ ተሳታፊዎች የኮሮና በሽታ መከላከያዎችን የመተግበር ልምምዶችን የተመለከቱ ጥያቄዎች**  ***መመሪያ፡ ከታች ለቀረቡት ጥያቄዎች፡ የእርስዎን ሃሳብ የሚገልፀው መልስ ላይ ካከበቡ በኃላ፡ መልስዎን የያዘውን ቁጥር/ሮች በመጨረሻው የመልስ መስጫ ሳጥን ውስጥ ያስቀምጡ*** | | | | |
| **ቁ** | | **ጥያቄዎች/ተለዋዋጮች** | **የመልስ አማራጮች** | **የመልስ መስጫ ሳጥን** |
|  | | ማስክ (የአፍና አፍንጫ መሸፈኛ) የኮሮና በሽታን ይከላከላል ብለህ/ሽ ታምናለህ/ሽ? | 1. አዎ 2. አላምንም |  |
|  | | ማስክ (የአፍና አፍንጫ መሸፈኛ) ሁልጊዜ ታደርጋለህ/ሽ? | 1. አዎ 2. ሁልጊዜ አላደርግም |  |
|  | | ማስክ ሁልጊዜ የማታደርግ ከሆነ፡ ምክንያትህ/ሽ ምንድነው?  ***(ከአንድ በላይ መልስ መምረጥ ይቻላል)*** | 1. ምቾት ስለማይሰጠኝ 2. በዙሪያዬ ያሉ ብዙ ሰዎች ስለማያደርጉ ከነሱ ላለመለየት 3. መግዛት ስለማልችል 4. ማስክ የኮሮና በሽታን ይከላከላል ብዬ ስለማላምን 5. የኮሮና በሽታ አለ ብዬ ስለማላምን 6. በተፈጥሮ አስተማማኝ በሽታን የመከላከል አቅም አለኝ ብዬ ስለማስብ 7. በኮሮና በሽታ የመያዝ ስጋት ስለሌለብኝ ወይም ለበሽታው ተጋላጭ ነኝ ብዬ ስለማላስብ 8. በሽታው ቢይዘኝም በቀላሉ እቋቋመዋለሁ (ጉዳት ኤደርስብኝም) ብዬ ስለማስብ 9. ስለማስክ ጥቅም በቂ ግንዛቤ/ዕውቀት ስለሌለኝ 10. ከሃይማኖት/ዕምነት ጋር የተያያዘ ምክንያት ስላለኝ 11. በምኖርበት/በምሰራበት አካባቢ የኮሮና በሽታ አሳሳቢ ስላልሆነ 12. ሌላ ምክንያት 13. ምክንያት የለኝም |  |
|  | | ለሁለተኛው ጥያቄ መልስዎ አዎ ከሆነ፡ የማስክ ንፅህና ሁልጊዜ ትጠብቃለህ/ሽ? | 1. አዎ 2. አልጠብቅም |  |
|  | | ማንኛውንም ዕቃ ከነኩ በኃላ፡ እጅን ቶሎ መታጠብ/ማፅዳት የኮሮና በሽታን ይከላከላል ብለህ/ሽ ታምናለህ/ሽ? | 1. አዎ 2. አላምንም |  |
|  | | ማንኛውንም ዕቃ ከነካህ/ሽ በኃላ፡ እጅህን ቶሎ በውሃ እና ሳሙና ትታጠባለህ//ሽ? | 1. አዎ 2. አልታጠብም/አላፀዳም |  |
|  | | ከላይ ለቀረበው ጥያቄ መልስዎ አልታጠብም ከሆነ፡ ምክንያትህ/ሽ ምንድነው?  ***(ከአንድ በላይ መልስ መምረጥ ይቻላል)*** | 1. ውሃና ሳሙና በአቅራቢያ አለማግኘት 2. ማንኛውንም ዕቃ ከመንካት አስቀድሜ ስለምጠነቀቅ 3. የኮሮና በሽታ አለ ብዬ ስለማላምን 4. የዕጅ ንፅህናን መጠበቅ የኮሮና በሽታን ይከላከላል ብዬ ስለማላምን 5. እጄን በአልኮል/ሳኒታይዘር ስለማፀዳ 6. በተፈጥሮ አስተማማኝ በሽታን የመከላከል አቅም አለኝ ብዬ ስለማስብ 7. በኮሮና በሽታ የመያዝ ስጋት ስለሌለብኝ ወይም ለበሽታው ተጋላጭ ነኝ ብዬ ስለማላስብ 8. በሽታው ቢይዘኝም በቀላሉ እቋቋመዋለሁ (ጉዳት ኤደርስብኝም) ብዬ ስለማስብ 9. በቂ ግንዛቤ/ዕውቀት ስለሌለኝ 10. ከሃይማኖት/ዕምነት ጋር የተያያዘ ምክንያት ስላለኝ 11. በምኖርበት/በምሰራበት አካባቢ የኮሮና በሽታ አሳሳቢ ስላልሆነ 12. ሌላ ምክንያት 13. ምክንያት የለኝም |  |
|  | | ውሃና ሳሙና ማግኘት በማትችልበት ሁኔታ፣ ዕቃዎችን ከነካህ/ሽ በኃላ፡ እጅህን በሳኒታይዘር ታፀዳለህ/ሽ? | 1. አዎ 2. አላፀዳም |  |
|  | | ከላይ ለቀረበው ጥያቄ መልስዎ አላፀዳም ከሆነ ምክንያትህ/ሽ ምንድነው?  ***(ከአንድ በላይ መልስ መምረጥ ይቻላል)*** | 1. ሳኒታይዘር መግዛት ስለማልችል/ስለማላገኝ 2. ማንኛውንም ዕቃ ከመንካት አስቀድሜ ስለምጠነቀቅ 3. የኮሮና በሽታ አለ ብዬ ስለማላምን 4. ሳኒታይዘር የኮሮና በሽታን ይከላከላል ብዬ ስለማላምን 5. በተፈጥሮ አስተማማኝ በሽታን የመከላከል አቅም አለኝ ብዬ ስለማስብ 6. በኮሮና በሽታ የመያዝ ስጋት ስለሌለብኝ ወይም ለበሽታው ተጋላጭ ነኝ ብዬ ስለማላስብ 7. በሽታው ቢይዘኝም በቀላሉ እቋቋመዋለሁ (ጉዳት ኤደርስብኝም) ብዬ ስለማስብ 8. በቂ ግንዛቤ/ዕውቀት ስለሌለኝ 9. ከሃይማኖት/ዕምነት ጋር የተያያዘ ምክንያት ስላለኝ 10. በምኖርበት/በምሰራበት አካባቢ የኮሮና በሽታ አሳሳቢ ስላልሆነ 11. ሌላ ምክንያት 12. ምክንያት የለኝም |  |

ለሚከተሉት ጥያቄዎች፡ ድርጊቱን ዘውትር የሚያደርጉት ከሆነ “***ሁልጊዜ***”፣ አልፎ አልፎ ብቻ የሚያደርጉት ከሆነ ***“አልፎ አልፎ***”፣ ፈፅሞ የማያደርጉት ከሆነ ደግሞ “***በፍፁም***” የሚለውን መልስ በመምረጥ ይመልሱ

| ቁ. | ጥያቄዎች/ተለዋዋጮች | የመልስ አማራጮች | የመልስ መስጫ ሳጥን |
| --- | --- | --- | --- |
|  | ማስክ (የአፍና የአፍንጫ መሸፈኛ) ታደርጋለህ/ሽ? | 1. ሁልጊዜ 2. አልፎ አልፎ 3. በፍፁም |  |
|  | ማንኛውንም ዕቃ ከነካህ/ሽ? በኃላ እጅህን በምን ያህል ጊዜ ታፀዳለህ/ሽ? (በውሃና ሳሙና መታጠብ ወይም አልኮል መጠቀም) | 1. ሁልጊዜ 2. አልፎ አልፎ 3. በፍፁም |  |
|  | ሰዎች በሚሰበሰቡበት አካባቢ በቂ (እስከ 2 ሜትር) አካላዊ ርቀትን ትጠብቃለህ/ሽ? | 1. ሁልጊዜ 2. አልፎ አልፎ 3. በፍፁም |  |
|  | የማስክን/ሽን ንፅህና ትጠብቃለህ/ሽ? (ማጠብ ወይም አዲስ ገዝቶ መቀየር) | 1. ሁልጊዜ 2. አልፎ አልፎ 3. በፍፁም |  |
| 5. | በምታስልበት ወይም በምታስነጥስበት ጊዜ፡ አፍ እና አፍንጫህን በክንድህ ትሸፍናለህ/ሽ? | 1. ሁልጊዜ 2. አልፎ አልፎ 3. በፍፁም |  |
| 6. | የኮሮና በሽታ ምልክቶች ሲታዩብህ/ሽ ቤት ትቀመጣለህ/ሽ? (ከቤት አለመውጣት)? | 1. ሁልጊዜ 2. አልፎ አልፎ 3. በፍፁም |  |
| 7. | ከሰዎች ጋር ትጨባበጣለህ/ሽ? (በእጅ ንክኪ ሰላምታ ትለዋወጣለህ/ሽ?) | 1. ሁልጊዜ 2. አልፎ አልፎ 3. በፍፁም |  |
| 8. | ፊትን (አይን፣ አፍ እና አፍንጫን) ትነካካለህ/ሽ? | 1. ሁልጊዜ 2. አልፎ አልፎ 3. በፍፁም |  |
| 9. | ያልበሰሉ ምግቦችን (ጥሮ ስጋ፣ አታክልት የመሳሰሉትን) ትመገባለህ/ሽ? | 1. ሁልጊዜ 2. አልፎ አልፎ 3. በፍፁም |  |
| 10. | ሰዎች በብዛት በሚሰበሰቡባቸው (የአምልኮ፣ የገበያ፣ ስብሰባ የመሳሰሉት) ቦታዎች ትሳተፋለህ/ሽ? | 1. ሁልጊዜ 2. አልፎ አልፎ 3. በፍፁም |  |
